# A cross-cultural study translating and validating the COMPAT-SF pain questionnaire in Telugu, Bengali and Hindi

**DOI:** 10.1007/s12664-025-01737-z

**Published:** 2025-02-18

**Authors:** M. Unnisa, A. Agarwal, C. Peddapulla, V. Sharma, S. Midha, S. Jagannath, R. Talukdar, A. E. Phillips, M. Faghih, J. Windsor, S. S. Olesen, P. Garg, A. M. Drewes, L. Kuhlmann, On behalf of the International Pancreatic Pain Consortium

**Affiliations:** 1 Pancreas Research Group, Department of Medical Gastroenterology, Asian Institute of Gastroenterology Hospitals, Hyderabad 500 082, India; 2 Department of Gastroenterology, All India Institute of Medical Sciences, New Delhi 110 029, India; 3 Department of Medicine, Division of Gastroenterology, Hepatology, and Nutrition, University of Pittsburgh School of Medicine, Pittsburgh, PA USA; 4 Division of Gastroenterology and Hepatology, Department of Medicine, Johns Hopkins University School of Medicine, Baltimore, MD USA; 5 Surgical and Translational Research Centre, Faculty of Medical and Health Science, University of Auckland, Auckland, New Zealand; 6 Centre for Pancreatic Diseases & Mech-Sense, Department of Gastroenterology and Hepatology, Aalborg University Hospital, Mølleparkvej 4, 9000 Aalborg, Denmark; 7 Department of Clinical Medicine, Aalborg University, Aalborg, Denmark

**Keywords:** Chronic pancreatitis, Culture, India, Language, Pain, Pain measurement

## Abstract

**Background and Objectives:**

Chronic pancreatitis (CP) is a fibroinflammatory disease causing functional injury. Abdominal pain is the predominant symptom negatively impacting the quality of life. The Comprehensive Pain Assessment Tool (COMPAT-SF) questionnaire, designed and validated to assess pain in CP, was previously only available in English and Danish. Given the high prevalence of CP in India, translating and validating COMPAT-SF into different languages becomes crucial.

**Methods:**

The COMPAT-SF underwent translation into three Indian languages (Hindi, Telugu and Bengali) and was back-translated to English to ensure cross-cultural equivalence. Validation was conducted at two tertiary care centers in India. As Hindi is the most widespread language, bilingual CP patients answered the COMPAT-SF in Hindi and English at a three-week interval. All sub-group answers were compared with patient data from the US. Structural equation modeling and confirmatory factor analysis were employed for validation.

**Results:**

Total 64 patients (19 Hindi-speaking,15 Telugu and 30 Bengali) were included and compared with 91 English-speaking patients. Translation adequacy was confirmed with > 85% concordance. Despite modest Cronbach alpha values in reliability analysis, structural equation modeling demonstrated high consistency with the original COMPAT-SF study. Some cultural differences in responses were observed, but the responses were comparable overall. Confirmatory factor analysis on the pooled data indicated an acceptable model fit and the Hindi version showed good accordance with the English version.

**Conclusion:**

The translated COMPAT-SF versions proved to be valid and reliable pain assessment tools for CP patients. The study underscores the importance of addressing pain comprehensively.

**Graphical Abstract:**

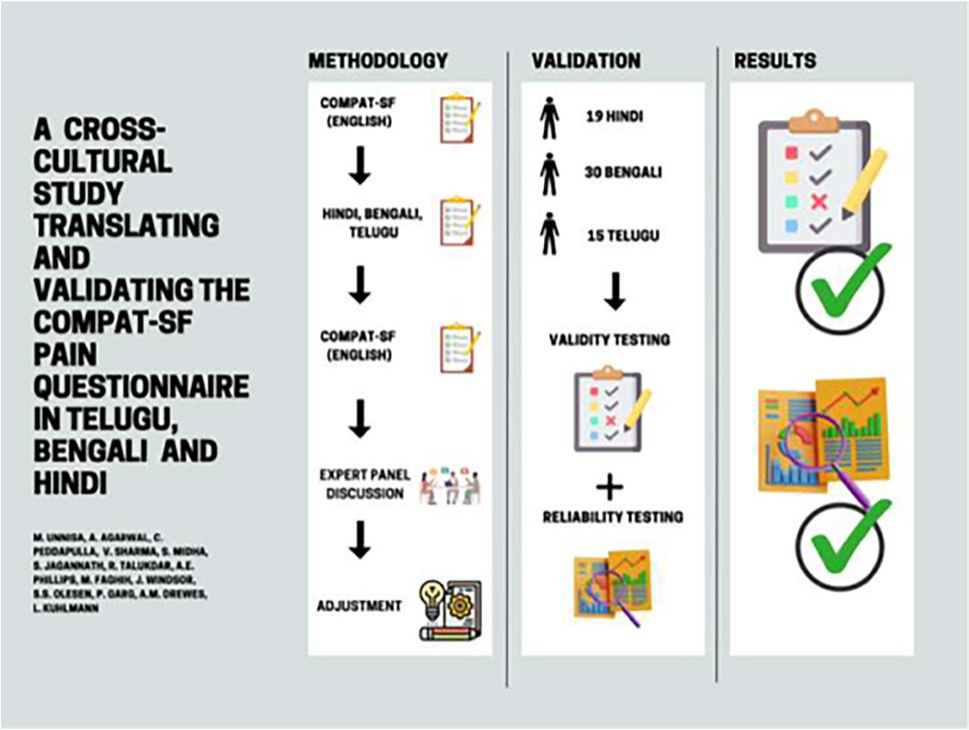

**Supplementary Information:**

The online version contains supplementary material available at 10.1007/s12664-025-01737-z.

## Introduction

Chronic pancreatitis (CP) is a fibro-inflammatory disease of the pancreas that results in functional injury [1]. It can impose significant morbidity associated with painful episodes that often result in frequent hospitalization [2]. Exocrine and endocrine insufficiency can further impair the quality of life. Pain remains a clinical challenge, as treatment is often difficult.

A comprehensive assessment of pain is essential for its treatment and response. Pain characteristics can also help guide the treatment type [1]. As a primary outcome in research, pain assessment needs to be able to detect changes over time [2]. Most tools for assessing pain are not comprehensive and have not been validated [3]. The recently developed ‘COMprehensive Pain Assessment Tool’ (COMPAT) aims to assess pancreatic pain thoroughly, focusing on the characteristics specific to pancreatic pain compared with somatic pain [4]. Although formal validation is ongoing, the short-form (COMPAT-SF) has been developed, separately validated and tested for reliability [5]. It is currently the only validated pain assessment tool for CP.

As the Indian population consists of more than 15% of the world’s population and the prevalence of CP is high [6], the need for Indian translations of COMPAT-SF is apparent. Furthermore, a large proportion of Indian CP patients are characterized as tropical pancreatitis patients, which show different characteristics than patients from other parts of the world [7]. These differences underline the importance of studying CP patients in India. Hindi (570 million), Bengali (97 million) and Telugu (81 million) are three of India’s most frequent first languages. Translation of the COMPAT-SF questionnaire into these languages will advance our understanding of pain and its assessment in patients with CP in India.

In this study, we aimed at translating the COMPAT-SF questionnaire into three common Indian languages (Hindi, Bengali and Telugu) and validate these to ensure the availability of a valid pain assessment tool for CP patients in India.

## Methods

### Questionnaire translation

The original English version of the COMPAT-SF questionnaire consisted of six questions divided into five pain dimensions: pain severity, pain fluctuation, pain provocation, spreading pain and qualitative pain descriptors.

A multi-faceted approach was adopted to achieve comprehensive translations and cross-cultural equivalence. Three native-speaking research scholars with in-depth knowledge of the English language were enlisted to translate the questionnaire. These scholars possessed an intimate understanding of the target languages and the associated cultural nuances and context-specific terminology. Their expertise allowed for meticulous and precise translations to maintain the questionnaire’s intended meaning and essence within the target language’s cultural context.

To validate the accuracy of the initial translations and enhance the overall quality of the process, three general English speakers, who were also proficient in the respective Indian languages, were engaged for back-translation. The selection of individuals proficient in both languages ensured their ability to effectively comprehend and articulate the nuances of both languages. The back-translation process served as a means of cross-validation, enabling the identification and rectification of any potential discrepancies or errors that may have arisen during the initial translation phase. The translation was considered adequate if there was > 85% concordance between the original and back-translated questionnaire and no consensus-evaluated significant linguistic losses in the parts lacking total concordance.

After completing the translation steps, the final translated versions of the COMPAT-SF questionnaire were named COMPAT-SF(T) (Indian Telugu), COMPAT-SF(B) (Indian Bengali) and COMPAT-SF(H) (Indian Hindi) (see Appendix A, B and C).

### Questionnaire validation

A prospective observational study was conducted between June 2022 and January 2023 at two tertiary care centers, Asian Institute of Gastroenterology, Hyderabad, India, and All India Institute of Medical Sciences, Delhi, India.

Inclusion criteria included age of at least 18 years, CP diagnosed from the Cambridge [8] or Rosemont [9] criteria from available imaging indicating definite CP, disease duration of at least three years and pancreatic pain, regardless of severity. Exclusion criteria included acute exacerbation of pancreatic pain within the last month, pregnancy and lactation, pancreatic and extrapancreatic malignancy, other painful conditions and major comorbidities such as chronic liver, kidney and cardiac diseases.

The questionnaire was administered to patients in their native language. As many Hindi-speaking Indians are bilingual, we chose to include only bilingual patients in this group to compare the Hindi version to the original version. The English version of the COMPAT-SF was administered after a timespan of three weeks after the Hindi questionnaire to diminish recollection bias. The timespan was not increased beyond this to reduce the risk of changes in pain patterns and characteristics due to the fluctuating nature of pain.

### Statistical analysis

Continuous data was expressed as mean with standard deviation (SD), while categorical data was expressed as proportions.

For validation, all group data was compared to the data from the original study in English (convergent validity) using a one-way analysis of variance (ANOVA) . The factor analysis from the structural equation modeling was also included as a validation method (construct validity). The Hindi-speaking group completed the questionnaire in both Hindi and English, as this group consisted of bilingual patients. Therefore, a direct comparison could be performed (concurrent validity).

Convergent validity was examined using one-way analysis of variance and pairwise comparison of means. A *p*-value < 0.05 was considered statistically significant.

To assess concurrent validity, the Hindi and English answers from the same patients were compared by calculating the two-way mixed-effects intra-class correlation coefficient (ICC) on single measurements. A paired *t*-test was used to examine differences in means and Bland Altman plots were made to visually inspect the limits of agreement. ICC values > 0.5 were considered moderate, > 0.75 good and > 0.9 excellent [10].

Reliability was assessed in several ways. Internal consistency was examined using Cronbach’s alpha, where values above 0.7 were considered acceptable. As Cronbach’s alpha might be underestimated due to the questionnaire’s structure, structural equation modeling was performed to evaluate model fit, including a path diagram and confirmatory factor analysis with goodness of fit indices. Chi-square, comparative fit index (CFI), Tucker-Lewis Index (TLI), standardized root mean square residual (SRMR) and root-mean-squared error of approximation (RMSEA) were evaluated. The goodness of fit was evaluated with the following cut-off values: *χ*^2^ *p*-value > 0.05, CFI > 0.95, TLI > 0.95, SRMR < 0.08 and RMSEA < 0.06 [11]. Multicollinearity was examined using variance inflation factor analysis, where values less than five were accepted [12].

Statistical analysis was performed using STATA version 16 (StataCorp LP, College Station, TX, US).

## Results

Seventy-eight patients were screened according to eligibility criteria and 64 were included. Fifteen patients completed the questionnaire in Telugu, 30 patients completed the questionnaire in Bengali and 19 patients completed it in Hindi. The answers were compared to 91 English-speaking patients (USA) from a previous study [5].

Demographical data and the mean questionnaire scores of the four groups are presented in Tables 1 and 2, respectively.

**Table 1:** Demographical data and questionnaire results

**Demographical data**					
	Hindi		Bengali	Telugu	English
Patients	19		30	15	91
Age, years	35.8 (18–64)		35.5 (19–67)	28.3 (18–49)	54.1 (19–86)
Male sex	11 (58)		17 (57)	10 (67)	52 (57)
Disease duration, years	7.6 (3–21)		6.93 (0–8)	2.7 (0–28)	7.5 (0–46)
Exocrine insufficiency	5 (26)		19 (63)	11 (73)	33 (36)
Diabetes	2 (11)		7 (47)	3 (20)	8 (9)
Average VAS score	4.9 (1–10)		5.4 (2–8)	5.2 (0–8)	6 (0–10)
Use of strong opioids	0 (0)		0 (0)	0 (0)	38 (42)
**Questionnaire results**					
	Hindi		Bengali	Telugu	English
	Hindi	English			
Severity score	51.9 (20–71)	53.2 (20–71)	58 (31–81)	58.5 (13–81)	56.8 (0–93)
Fluctuation score (constant)	6 (32)	8 (42)	17 (57)	11 (73)	48 (53)
Provocation score	14.9 (4–31)	12.7 (0–31)	14.2 (0–73)	21.1 (4–46)	23.6 (0–71)
Spreading score	23.7 (0–64)	22.2 (4–64)	28.6 (0–93)	22.1 (0–43)	22.8 (0–96)
Descriptive score	23.7 (5–59)	22.3 (0–55)	29.6 (1–89)	39.6 (0–68)	38 (0–81)
Total score	39.1 (24–60)	39.2 (25–57)	44.9 (25–80)	49.3 (31–66)	48.9 (19–80)

Values are presented as mean (SD)

*VAS* visual analogue scale

**Table 2:** Intra-class correlation coefficients between Hindi and English answers

Score	Intra-class correlation coefficient	95% confidence interval
Severity score	0.9336	[0.75–0.98]
Fluctuation score	0.5977	[0.14–0.93]
Provocation score	0.6734	[0.21–0.94]
Spreading score	0.7240	[0.31–0.94]
Descriptive score	0.9394	[0.71–0.99]

### Validity

**Fig. 1:**
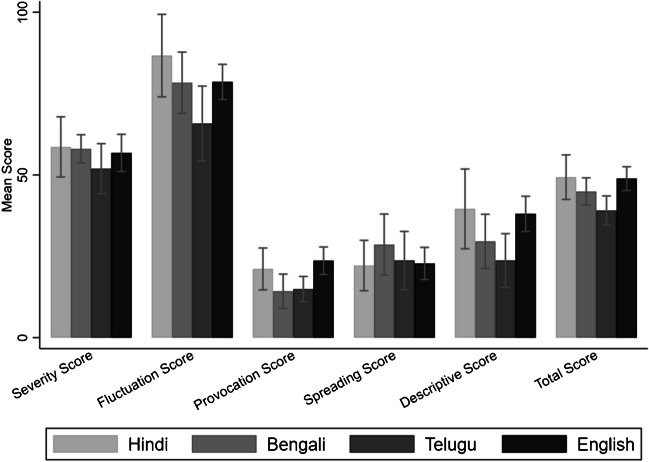
Pain dimension scores. This figure shows the differences in pain dimension scores across sub-groups

The convergent validity of the translated versions was evaluated by comparing the scores of patients who completed the questionnaire in their native language with those who completed it in English. The results showed no significant differences in all scores except the provocation score obtained from the English and native versions of all three languages. The Bengali answers differed significantly from the English answers in the provocation score (mean difference 9.4, *p* = 0.041). The values are presented in Fig. 1.

Construct validity was assessed using the structural equation modeling and the incorporated confirmatory factor analysis. The model indicated that pain severity (β = 1, *p* < 0.001), pain fluctuation (β = 1.18, *p* < 0.001), pain-provoking factors (β = 0.91, *p* < 0.001), spreading pain (β = 0.59, *p* < 0.014) and pain-describing adjectives (β = 2.22, *p* < 0.001) had significant positive effects on pain sensation. In addition, there were no signs of severe multicollinearity, with variance inflation factors varying from 1.09 to 1.44 and a mean of 1.26.

**Fig. 2:**
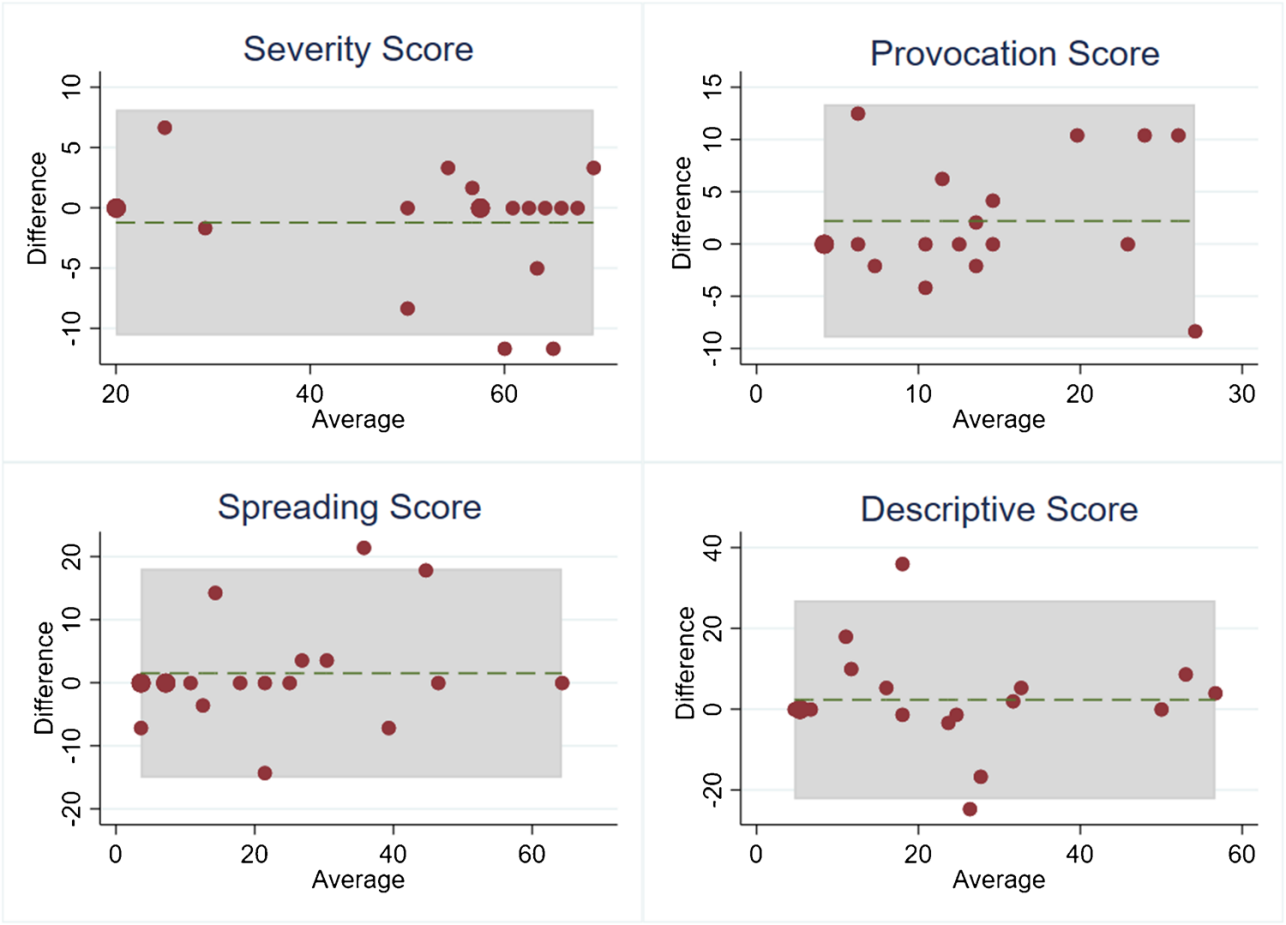
Bland-Altman Plots. Bland-Altman plots comparing the Hindi patients’ answers to the Hindi and the English questionnaire version. Dot sizes indicate the number of patients with the same value

The concurrent validity results comparing the Hindi and English answers of the bilingual patients showed no differences in mean scores with severity score *t* (17) = − 1.12, *p* = 0.28; fluctuation score *t* (17) = − 1.00, *p* = 0.33; provocation score *t* (17) = 1.64, *p* = 0.12; spreading score *t* (17) = 0.78, *p* = 0.45 and descriptive score *t* (17) = 0.79, *p* = 0.44. Intraclass correlation coefficients were calculated for all pain dimensions and varied from moderate to excellent (Table 3), with fluctuation score ICC as the lowest. Bland-Altman plots were drawn and inspected visually. Mean values were all close to zero and limits of agreement were acceptable in the context of the study (Fig. 2).

**Table 3:** Confirmatory factory analysis

**Confirmatory factor analysis**				
	Coefficient	*p*-value		*R*^2^
Severity score	1	< 0.001		0.22
Fluctuation score	0.32	< 0.001		0.20
Provocation score	0.22	< 0.001		0.27
Spreading score	0.24	< 0.014		0.07
Descriptive score	0.52	< 0.001		0.80
Overall				0.83
**Goodness of fit indices**				
	Value		*p*-value	
*χ*^2^	4.608		0.466	
RMSEA	< 0.001		0.636	
Comparative fit indices (CFI)	1.000			
Tucker-Lewis Index	1.009			
SRMR	0.040			

*RMSEA* root-mean-squared error of approximation, *SRMA* standardized root mean square residual

### Reliability

**Fig. 3:**
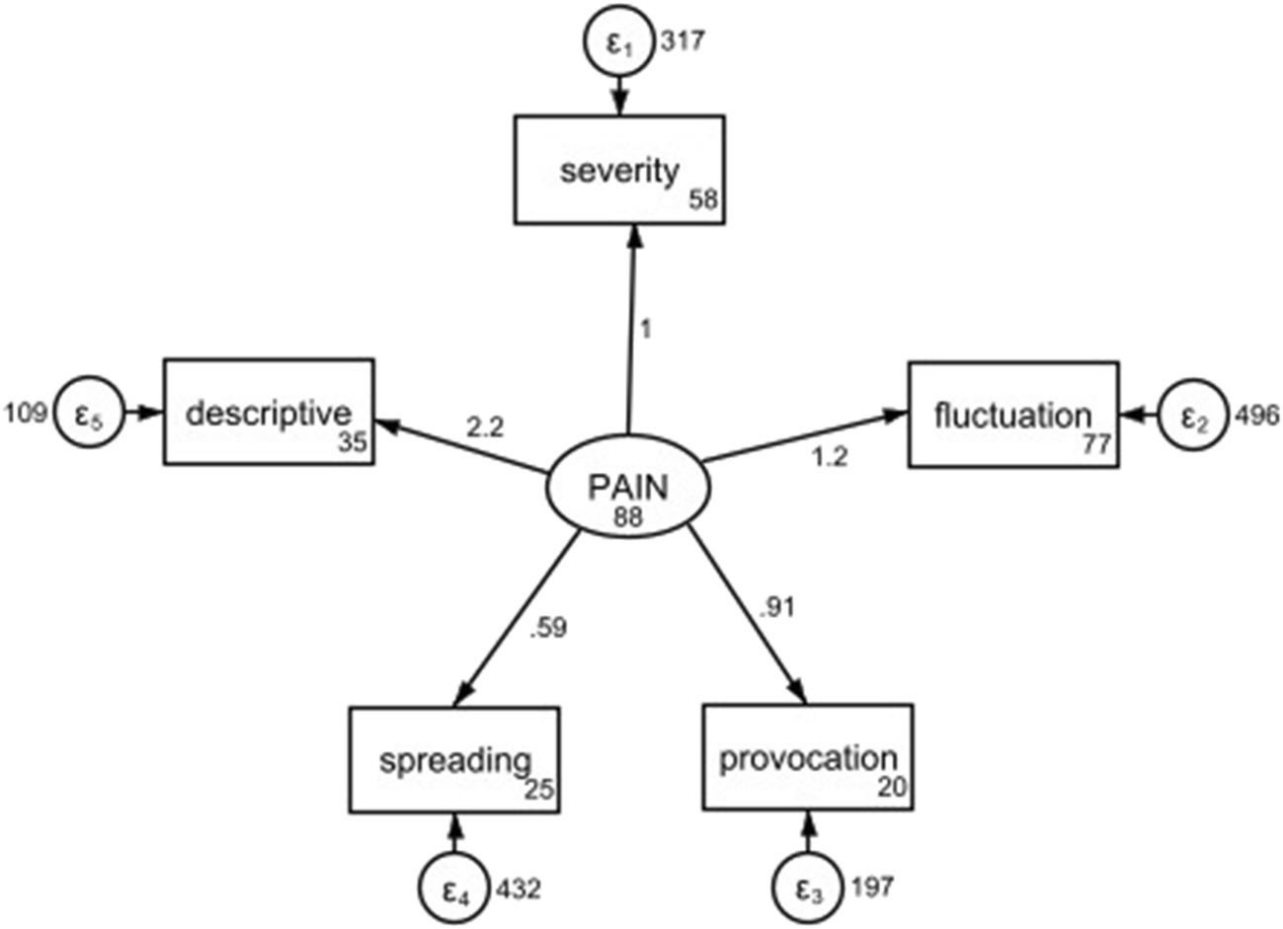
Structural equation modelling. Latent variables are shown in oval shapes and observed variables are in rectangles. Residuals are depicted as circles and loadings are added to the figure in numerical value

Cronbach alpha ranged from poor to moderate (Telugu = 0.75, Bengali = 0.53, Hindi = 0.60). Structural equation modeling was performed to shed light on the relationship between the questions. Due to the small sample sizes, structural equation modeling was performed with pooled data from all sub-groups (Fig. 3). In the proposed model, all pain dimensions affected the general sensation of pain. According to the goodness of fit indices, the model fitted the data well, *χ*^2^(5) = 4.608, *p* = 0.466, CFI = 1.000, TLI = 1.009, RMSEA < 0.001, SRMR = 0.04 (Table 3).

## Discussion

In this prospective observational study, we translated COMPAT-SF into three regional languages (Telugu, Bengali and Hindi). The translation was done meticulously, following available guidelines [13]. Reliability analysis showed a moderate Cronbach alpha value, but confirmatory analysis of the data compiled with the original COMPAT-SF cohort from the original COMPAT-SF study showed that the data fit the questionnaire model well. Validity testing showed the questionnaire was valid, correlating answers in most categories and scores. Confirmatory factor analysis showed that all proposed dimensions impacted the sensation of pain and concurrent validity analysis showed that the Hindi version of the questionnaire aligned with the English version.

We have calculated Cronbach alpha for all three translated questionnaires and found it to range from poor to moderate. The varying Cronbach alpha is similar to other translated pain questionnaires [14]. The COMPAT-SF assesses different dimensions of pain, which are believed to be interrelated. However, the questions are not unidimensional, as the concept of pain is wide-ranging and multidimensional. The low variance inflation factor also supports this hypothesis. This means that the reliability will tend to be underestimated when evaluated by Cronbach alpha, as shown [15]. We, therefore, added the structural equation model as a comparative method of estimating reliability, adding the original data from the COMPAT-SF study [5] and this showed that the questionnaires were consistent with what was previously shown.

The validity was assessed by exploring both convergent and construct validity. We also assessed concurrent validity for the Hindi population due to bilingualism.

The convergent validity showed high correlations between most scores. As subjective features of pain are highly culturally dependent [16] and lifestyle, as well as environmental risk factors for pain, differ between the original American cohort and the Indian cohort, we believe that the statistical differences found in questions concerning pain-provoking factors and pain are influenced by cultural rather than translational differences [17]. The differences might also be explained by the age difference between the English and Indian cohorts, as age can influence pain characteristics, as higher age is associated with less interference, less catastrophizing and differences in qualitative description [18, 19]. This might also explain the relatively low coefficients of the confirmatory factor analysis. However, the goodness of fit indices indicates that the translations are good and valid.

Concurrent validity analysis showed that patients answered the Hindi and English versions similarly. ICC was only moderate for the pain fluctuation group, but a contributing factor could be changes in pain patterns, as questionnaires were answered three weeks apart. Changes in pain characteristics were an apparent risk when choosing to separate the answers to the Hindi and the English questionnaires by weeks [20]. However, the risk of overestimating validity due to recall was of greater concern [21].

Methodologically, this study included a questionnaire translation that rigorously adheres to accepted standards, including the crucial step of back-translation to ensure equivalence [22]. This meticulous process establishes a foundation for basic validity. While back-translation in questionnaire translations is becoming more prevalent, historical practices have not consistently embraced this method. Contemporary trends, however, indicate an increasing adoption of back-translation, enhancing the validity of translated instruments and ensuring linguistic validation [14, 23]. Notably, many translated questionnaires often lack validation beyond the linguistic aspect. For instance, when scrutinizing the widely used pain assessment scale, the Brief Pain Inventory, more than half of the translated versions lack validation beyond linguistic validation [24].

To strengthen the validation process, it is important to include psychometric validation. In this study, we comprehensively validated the Indian versions of the COMPAT-SF, encompassing convergent, construct and concurrent validity. The selection of validity testing methods is seldom decisive, recognizing that no single method is foolproof [22]. While various forms of factor analysis are commonly employed in validating translated instruments [14, 25], our approach expanded the dimensions of validity assessment to ensure complete validation, ensuring the ability to compare with other validations of translated pain assessment instruments. Notably, most studies are constrained to two validation methods and we included three methods [14, 26].

Concurrent validity, often considered a gold standard, involves comparing the new questionnaire to the original version. Acknowledging the challenge of finding sufficient bilingual patients, we posit that concurrent validity is evident for the Hindi questionnaire, suggesting its probable extension to the other Indian language versions [27].

The study exhibits a combination of strengths and limitations. The study’s strengths lie in its comprehensive and meticulous translation process, adhering rigorously to international standards to ensure linguistic validity. Another strength is the multi-faceted validation process encompassing various levels to assess the reliability and validity of the translated instruments robustly.

Limitations include the constrained sample size within each language cohort, which consisted of 64 patients with 15-30 individuals in each group.

Guidelines for sample size calculation in questionnaire translation studies recommend a respondent-item ratio ranging from 5:1 to 30:1 [28, 29]. In this study, the respondent-item ratio varied between 3:1 and 6:1 in the different sub-groups, with 12:1 in the total group. Insufficient power in the study raises the potential for a type-II error, increasing the likelihood of overlooking relationships between variables.

Secondly, concurrent validity was not assessed in the Bengali and Telugu cohorts because of the scarcity of proficient bilingual patients. This limitation underscores the importance of addressing linguistic complexities in future research.

Crucially, the three Indian languages selected for this study represent only a fraction of the diverse national languages in India. To comprehensively establish the validity of the COMPAT-SF in India, translation and validation across all languages spoken in the country would be required. However, the chosen languages, being among the most widely used, demonstrate observed validity, suggesting that future translations into other Indian languages may not necessitate additional cultural adaptation and are likely to maintain validity.

Emphasizing the need for ongoing validation efforts as more data becomes available is essential. Continuous evaluation will contribute to refining and augmenting the study’s findings, ensuring their sustained relevance and applicability over time.

To conclude, the translation and adaptation of the Comprehensive Pain Assessment Tool (COMPAT-SF) into Hindi, Telugu and Bengali represent a significant step in facilitating comprehensive pain assessment in patients with painful CP within diverse linguistic and cultural contexts. These translated versions provide healthcare professionals and researchers in India and beyond with valuable tools to assess and manage pain effectively among speakers of these languages. It underscores the importance of addressing pain comprehensively in diverse populations, with the potential to improve the quality of life and patient outcomes in these linguistic and cultural contexts.

## Supplementary Information

Below is the link to the electronic supplementary material.

Supplementary file1 (DOCX 95 KB)

Supplementary file2 (DOCX 484 KB)

Supplementary file3 (PDF 306 KB)

## Data Availability

Upon request.

## Declarations

### Disclaimer

The authors are solely responsible for the data and the contents of the paper. In no way, the Honorary Editor-in-Chief, Editorial Board Members, the Indian Society of Gastroenterology or the printer/publishers are responsible for the results/findings and content of this article.

### Disclaimer

The authors are solely responsible for the data and the content of the paper. In no way, the Honorary Editor-in-Chief, Editorial Board Members, the Indian Society of Gastroenterology or the printer/publishers are responsible for the results/findings and content of this article.
