# Supplementary material for: A cross-cultural study translating and validating the COMPAT-SF pain questionnaire in Telugu, Bengali and Hindi
Source: Indian J Gastroenterol. 2025 Feb 18;44(5):684–91. doi: 10.1007/s12664-025-01737-z (PMC12417265; doi:10.1007/s12664-025-01737-z)
Supplement: Supplementary file 1 — Supplementary file1 (DOCX 95 KB) [file 12664_2025_1737_MOESM1_ESM.docx]

**Appendix A-Telugu:** దీర్ఘకాలిక ప్యాంక్రియాటైటిస్ కోసం సమగ్ర నొప్పి అసెస్‌మెంట్ టూల్-షార్ట్ ఫారమ్ **(COMPAT-SF):**

తేదీ:_________

పేరు: రోగి ఐది: లింగము:

జాతి: వయస్సు: వృత్తి:

ఈ అధ్యయనంలో పాల్గొన్నందుకు ధన్యవాదాలు. దయచేసి దిగువ మీ వివరాలను నింపండి లేదా రోగి లేబుల్ అతికించండి.

*సూచనలు: దీర్ఘకాలిక పాంక్రియాటైటిస్ లో మీ ప్యాంక్రియాటిక్ నొప్పి అనుభవం గురించి దిగువ ప్రశ్నలు అడగబడ్డాయి. ఇది సాధారణంగా పొత్తికడుపు పై భాగంలో ఎక్కడో అనుభూతి చెందుతుంది.*

*Q1. దయచేసి గత 12 నెలల్లో మీ నొప్పి అనుభవాన్ని ఉత్తమంగా సూచించే ప్యాంక్రియాటిక్ నొప్పి నమూనాను సర్కిల్ చేయండి.*

భరించలేని నొప్పి

నొప్పి లేదు a.మధ్యలనొప్పి లేకుండా b. నొప్పి దాడులతో నిరంతర నొప్పి

నొప్పి దాడులు

c. రోజువారీ స్థిరమైన నొప్పి d. మధ్యలో తగ్గిన నొప్పి విరామాలతో

తీవ్రమైన నిరంతర నొప్పి

ప్ర2. గత 12 నెలల కాలంలో నొప్పి తీవ్రత.

*దయచేసి ఈ ప్రతి స్కేల్ స్కేలుపై ఒక ఎక్స్ ఉంచండి.*

**1.నొప్పి యొక్క**

**సగటు తీవ్రత**  **0 1 2 3 4 5 6 7 8 9 10**

**నొప్పి మితమైన భరించలేని**

**లేదు నొప్పి నొప్పి**

**2. నొప్పి యొక్క**

**భరించలేని** **0 1 2 3 4 5 6 7 8 9 10**

**తీవ్రత** **నొప్పి మితమైన భరించలేని**

**లేదు నొప్పి నొప్పి**

**3.నొప్పి యొక్క**

**కనీసం తీవ్రత**  **0 1 2 3 4 5 6 7 8 9 10**

**నొప్పి మితమైన భరించలేని**

**లేదు నొప్పి నొప్పి**

ప్ర3. దయచేసి మీ ప్రస్తుత నొప్పి మందులు మరియు మోతాదును క్రింద వ్రాయండి మరియు ఫ్రీక్వెన్సీ కోసం మీ సమాధానాలను వృత్తం చుట్టండి

ఔషధ మోతాదు పౌనఃపున్యం

____________________________________________________________________________

పిఆర్ఎన్ / ఓడి / బిడి / టిడిఎస్ / క్యూఐడి

పిఆర్ఎన్ / ఓడి / బిడి / టిడిఎస్ / క్యూఐడి

పిఆర్ఎన్ / ఓడి / బిడి / టిడిఎస్ / క్యూఐడి

పిఆర్ఎన్ / ఓడి / బిడి / టిడిఎస్ / క్యూఐడి

పిఆర్ఎన్ / ఓడి / బిడి / టిడిఎస్ / క్యూఐడి

పిఆర్ఎన్ / ఓడి / బిడి / టిడిఎస్ / క్యూఐడి

______________________________________________________________________________________________________________________________________________________

పిఆర్ఎన్: అవసరమైనప్పుడు; ఓడి: రోజూ ఒకసారి; బిడి: రోజూ రెండుసార్లు; టిడిఎస్: రోజూ మూడుసార్లు; క్యూఐడి: రోజూ నాలుగుసార్లు.

ప్ర4. మీ ప్యాంక్రియాటిక్ నొప్పిని తీసుకువచ్చే ప్రతి ఐటమ్ కు దయచేసి రేటింగ్ ఇవ్వండి.

అంశాలు ఎన్నడూ అరుదుగా కొన్నిసార్లు చాలా తరచుగా ఎల్లప్పుడూ వర్తించదు

1. ఏదైనా ఆహారం ☐ ☐ ☐ ☐ ☐ ☐

2. కొవ్వు ఆహారం ☐ ☐ ☐ ☐ ☐ ☐

3. ద్రవాలు తాగడం ☐ ☐ ☐ ☐ ☐ ☐

4. మద్యం సేవించడం ☐ ☐ ☐ ☐ ☐ ☐

5. ఒత్తిడి ☐ ☐ ☐ ☐ ☐ ☐

6. సిగరెట్ ధూమపానం ☐ ☐ ☐ ☐ ☐ ☐

7. వ్యాయామం ☐ ☐ ☐ ☐ ☐ ☐

8. సామాజికంగా ☐ ☐ ☐ ☐ ☐ ☐ ఉండటం

9. కాలము మార్పులు ☐ ☐ ☐ ☐ ☐ ☐

10. చర్మంపై ☐ ☐ ☐ ☐ ☐ ☐ తేలికపాటి స్పర్శ

11. చర్మంపై ☐ ☐ ☐ ☐ ☐ ☐ జలుబు/వేడి

12. చర్మంపై ఒత్తిడి ☐ ☐ ☐ ☐ ☐ ☐

13. ఇతరులు ☐ ☐ ☐ ☐ ☐ ☐ (దయచేసి పేర్కొనండి):

ప్ర5. మీ సాధారణ ప్యాంక్రియాటిక్ నొప్పితో పాటు, మీరు అనుభవించే ప్రతి ఐటమ్ కు దయచేసి రేటింగ్ ఇవ్వండి.

అంశాలు ఎన్నడూ అరుదుగా కొన్నిసార్లు చాలా తరచుగా ఎల్లప్పుడూ

1. తల మరియు/లేదా ముఖ నొప్పి ☐ ☐ ☐ ☐ ☐

2. కీళ్ల నొప్ప ☐ ☐ ☐ ☐ ☐

3. చేతులు/కాళ్ళు నొప్పి ☐ ☐ ☐ ☐ ☐

4. వెన్ను మరియు/లేదా మెడ నొప్పి ☐ ☐ ☐ ☐ ☐

(క్లోమం నొప్పికి సంబంధించినది కాదు)

5. పొత్తికడుపు మరియు/లేదా కటి నొప్పి ☐ ☐ ☐ ☐ ☐

(కాదు చెందిన కు క్లోమం నొప్పి)

6. కండరాల నొప్పి ఉదా: ☐ ☐ ☐ ☐ ☐

ఫైబ్రోమయాల్జియా

7. ఛాతీ నొప్పి ☐ ☐ ☐ ☐ ☐

8. ఇతరులు (దయచేసి పేర్కొనండి): ☐ ☐ ☐ ☐ ☐

ప్ర6. నొప్పి మరియు సంబంధిత లక్షణాల యొక్క కొన్ని విభిన్న లక్షణాలను వివరించే పదాల జాబితా దిగువన ఇవ్వబడింది. గత 12 నెలల కాలంలో మీరు అనుభవించిన ప్రతి నొప్పి మరియు సంబంధిత లక్షణాల యొక్క తీవ్రతను అత్యుత్తమంగా వివరించే నెంబర్లను దయచేసి వృత్తం చుట్టండి. ఒకవేళ పదం మీ నొప్పి లేదా సంబంధిత లక్షణాలను వివరించనట్లయితే 0 ఉపయోగించండి.

| 1.నొప్పి పుడుతోంది | నొప్పి లేదు | 0 | 1 | 2 | 3 | 4 | 5 | 6 | 7 | 8 | 9 | 10 | భరించలేని నొప్పి |
| --- | --- | --- | --- | --- | --- | --- | --- | --- | --- | --- | --- | --- | --- |
| 2. షూటింగ్ నొప్పి | నొప్పి లేదు | 0 | 1 | 2 | 3 | 4 | 5 | 6 | 7 | 8 | 9 | 10 | భరించలేని నొప్పి |
| 3. కత్తిపోటు నొప్పి | నొప్పి లేదు | 0 | 1 | 2 | 3 | 4 | 5 | 6 | 7 | 8 | 9 | 10 | భరించలేని నొప్పి |
| 4. పదునైన నొప్పి | నొప్పి లేదు | 0 | 1 | 2 | 3 | 4 | 5 | 6 | 7 | 8 | 9 | 10 | భరించలేని నొప్పి |
| 5. తిమ్మిరి నొప్పి | నొప్పి లేదు | 0 | 1 | 2 | 3 | 4 | 5 | 6 | 7 | 8 | 9 | 10 | భరించలేని నొప్పి |
| 6. కొరికే నొప్పి | నొప్పి లేదు | 0 | 1 | 2 | 3 | 4 | 5 | 6 | 7 | 8 | 9 | 10 | భరించలేని నొప్పి |
| 7. వేడిమి తో మండుతున్న నొప్పి | నొప్పి లేదు | 0 | 1 | 2 | 3 | 4 | 5 | 6 | 7 | 8 | 9 | 10 | భరించలేని నొప్పి |
| 8. నొప్పి నొప్పి | నొప్పి లేదు | 0 | 1 | 2 | 3 | 4 | 5 | 6 | 7 | 8 | 9 | 10 | భరించలేని నొప్పి |
| 9. భారీ నొప్పి | నొప్పి లేదు | 0 | 1 | 2 | 3 | 4 | 5 | 6 | 7 | 8 | 9 | 10 | భరించలేని నొప్పి |
| 10. లేతగా | నొప్పి లేదు | 0 | 1 | 2 | 3 | 4 | 5 | 6 | 7 | 8 | 9 | 10 | భరించలేని నొప్పి |
| 11. నొప్పిని చీల్చడం | నొప్పి లేదు | 0 | 1 | 2 | 3 | 4 | 5 | 6 | 7 | 8 | 9 | 10 | భరించలేని నొప్పి |
| 12. అలసట-అలసట | నొప్పి లేదు | 0 | 1 | 2 | 3 | 4 | 5 | 6 | 7 | 8 | 9 | 10 | భరించలేని నొప్పి |
| 13. అనారోగ్యకరమైన | నొప్పి లేదు | 0 | 1 | 2 | 3 | 4 | 5 | 6 | 7 | 8 | 9 | 10 | భరించలేని నొప్పి |
| 14. భయం | నొప్పి లేదు | 0 | 1 | 2 | 3 | 4 | 5 | 6 | 7 | 8 | 9 | 10 | భరించలేని నొప్పి |
| 15. శిక్షించే-క్రూరమైన | నొప్పి లేదు | 0 | 1 | 2 | 3 | 4 | 5 | 6 | 7 | 8 | 9 | 10 | భరించలేని నొప్పి |

మీరు పాల్గొన్నందుకు ధన్యవాదాలు.
