# Supplementary material for: A cross-cultural study translating and validating the COMPAT-SF pain questionnaire in Telugu, Bengali and Hindi
Source: Indian J Gastroenterol. 2025 Feb 18;44(5):684–91. doi: 10.1007/s12664-025-01737-z (PMC12417265; doi:10.1007/s12664-025-01737-z)
Supplement: Supplementary file 2 — Supplementary file2 (DOCX 484 KB) [file 12664_2025_1737_MOESM2_ESM.docx]

**Appendix B-Bengali: ক্রনিক প্যানক্রিয়াটাইটিসের জন্য কম্প্রিহেনসিভ পেইন অ্যাসেসমেন্ট টুল-শর্ট ফর্ম (COMPAT-SF)**

তারিখ:

রোগীর নাম: রোগীর সংখ্যা:

জাতিগত: বয়স:

এই গবেষণায় অংশ নেওয়ার জন্য আপনাকে ধন্যবাদ। অনুগ্রহ করে নীচে আপনার বিবরণগুলি পূরণ করুন বা একটি রোগীর লেবেল সংযুক্ত করুন।

*নির্দেশাবলী: ক্রনিক প্যানক্রিয়াটাইটিসে আপনার অগ্ন্যাশয়ের ব্যথার অভিজ্ঞতা সম্পর্কে নিম্নলিখিত প্রশ্নগুলি জিজ্ঞাসা করা হয়। এটি সাধারণত পেটের উপরের অংশে কোথাও অনুভূত হয়।*

Q1। অনুগ্রহ করে অগ্ন্যাশয়ের ব্যথার প্যাটার্নটি বৃত্ত করুন যা গত 12 মাসে আপনার ব্যথার অভিজ্ঞতাকে সর্বোত্তমভাবে উপস্থাপন করে।

সবচেয়ে খারাপ ব্যথা


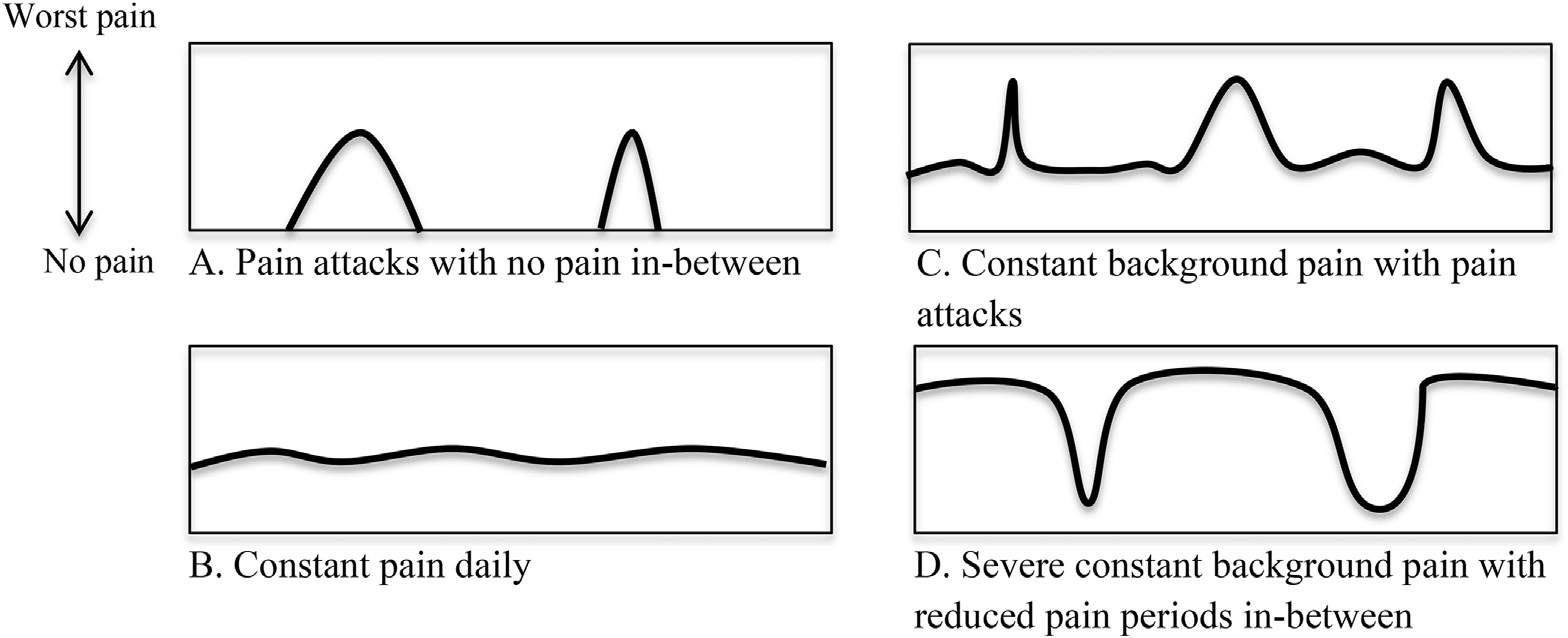


D. তীব্র ধ্রুবক পটভূমি ব্যথা কম ব্যথা পিরিয়ড সঙ্গে মধ্যে মধ্যে

মধ্যে কোন ব্যথা সঙ্গে ব্যথা আক্রমণ

মধ্যে কোন ব্যথা সঙ্গে ব্যথা আক্রমণ

প্রতিদিনের ব্যথা

C. ব্যথা আক্রমণ সঙ্গে ধ্রুবক পটভূমি ব্যথা

কোনও ব্যথা নেই

Q2। গত 12 মাস ধরে ব্যথার তীব্রতা ।


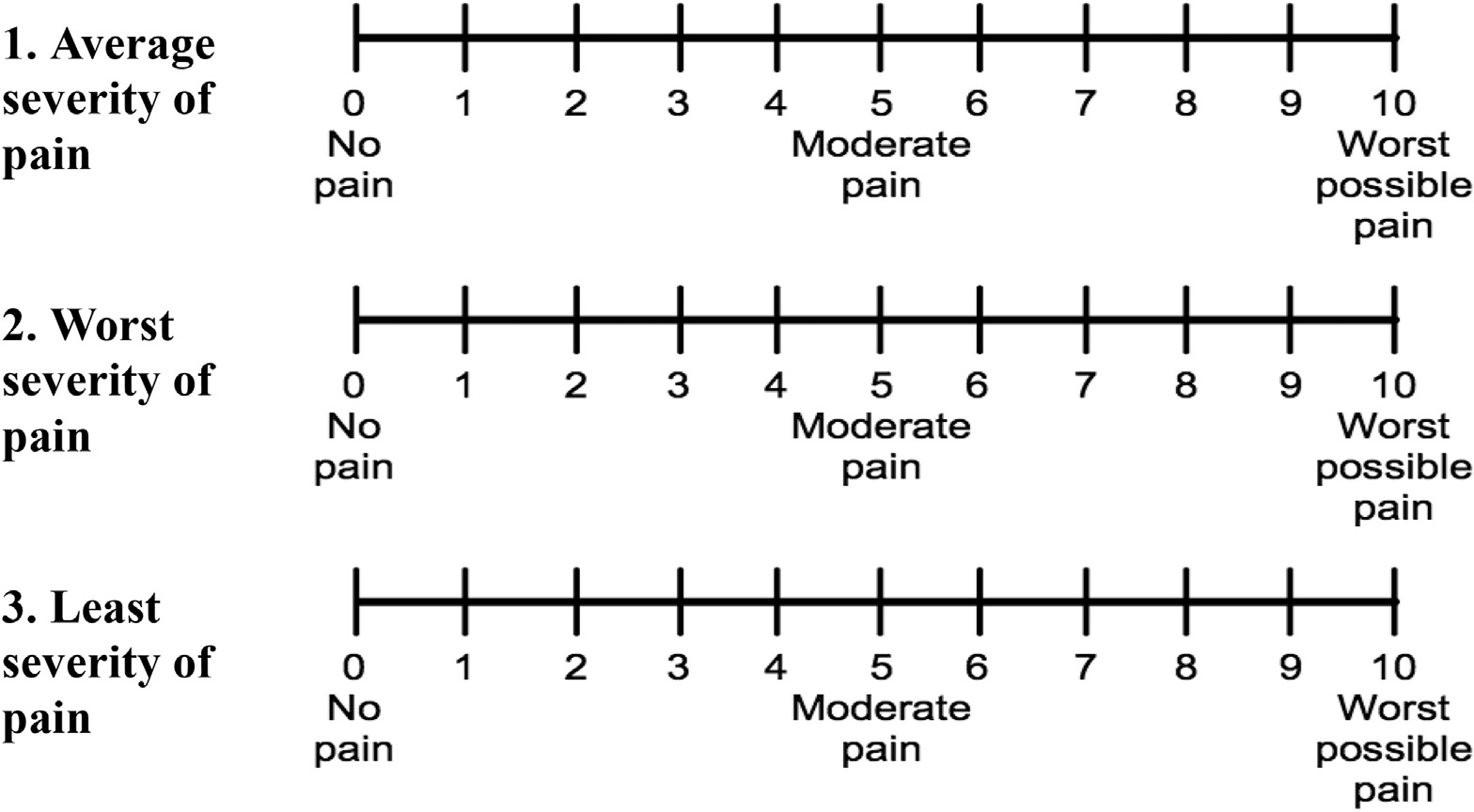
*অনুগ্রহ করে এই স্কেলগুলির প্রতিটিতে একটি X রাখুন*

সবচেয়ে খারাপ ব্যথা

মাঝারি ব্যথা

কোনও ব্যথা নেই

সবচেয়ে খারাপ ব্যথা

মাঝারি ব্যথা

কোনও ব্যথা নেই

মাঝারি ব্যথা

কোনও ব্যথা নেই

সবচেয়ে খারাপ ব্যথা

**3. ব্যথার সর্বনিম্ন তীব্রতা**

**2. ব্যথার সবচেয়ে খারাপ তীব্রতা**

**1. ব্যথার গড় তীব্রতা**

Q3। অনুগ্রহ করে নীচে আপনার বর্তমান ব্যথার ওষুধ এবং ডোজ লিখুন এবং ফ্রিকোয়েন্সির জন্য আপনার উত্তরগুলি বৃত্তাকার করুন

ঔষধ ডোজ ফ্রিকোয়েন্সি

PRN / OD / BD / TDS / QID

PRN / OD / BD / TDS / QID

PRN / OD / BD / TDS / QID

PRN / OD / BD / TDS / QID

PRN / OD / BD / TDS / QID

|  | PRN / OD / BD / TDS / QID |
| --- | --- |
|  |  |

PRN: যখন প্রয়োজন হয়, OD: একবার দৈনিক, BD: দুইবার দৈনিক, TDS: তিনবার দৈনিক, QID: দৈনিক চারবার

Q4। অনুগ্রহ করে আপনার অগ্ন্যাশয়ের ব্যথা নিয়ে আসা প্রতিটি আইটেমকে রেট দিন।


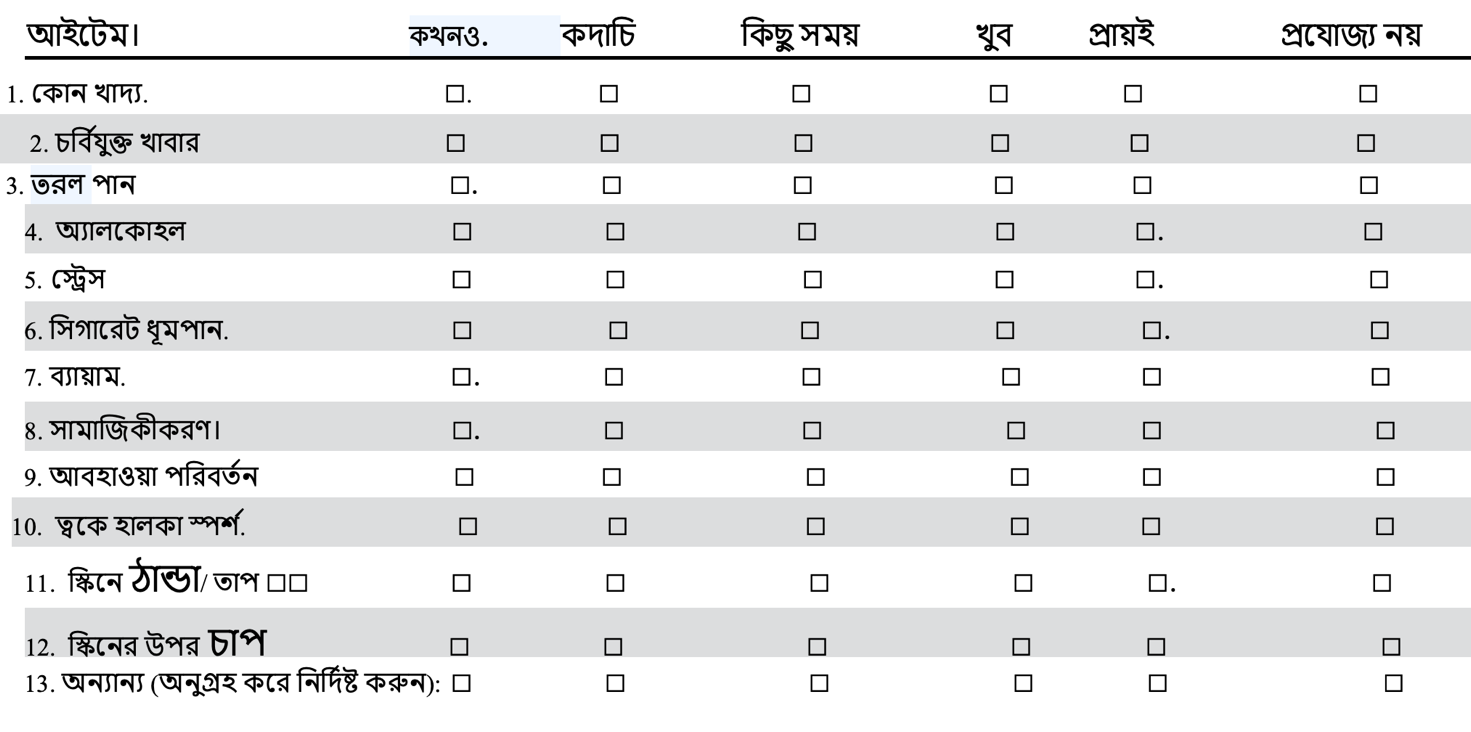


যে কোন খাদ্য

Q5। আপনার সাধারণ অগ্ন্যাশয়ের ব্যথা ছাড়াও, দয়া করে আপনার অভিজ্ঞতার প্রতিটি আইটেমকে রেট দিন

বিষয়োপকরণ কখনও কদাচিত্ কখনও কখনও প্রায়শই সর্বদা

1. মাথা এবং / অথবা ফেসিয়াল ব্যাথা। ☐ ☐ ☐ ☐ ☐

2. জয়েন্টে ব্যথা ☐ ☐ ☐ ☐ ☐

3. উপরের এবং / অথবা / অথবা / নিম্ন অঙ্গ ব্যথা ☐ ☐ ☐ ☐ ☐

4. পিছনে এবং / অথবা ঘাড় ব্যথা ( অগ্ন্যাশয় ব্যথা সম্পর্কিত নয় ) ☐ ☐ ☐ ☐ ☐

5. পেট এবং/অথবা শ্রোণী ব্যাথা। (নয়) সম্পর্কিত করতে অগ্ন্যাশয় ব্যথা) ☐ ☐ ☐ ☐ ☐

6. পেশী ব্যথা উদাঃ ফাইব্রোমায়ালজিয়া ☐ ☐ ☐ ☐ ☐

7. বুকে ব্যথা ☐ ☐ ☐ ☐ ☐

8. অন্যান্য (অনুগ্রহ করে নির্দিষ্ট করুন): ☐ ☐ ☐ ☐ ☐

Q6। নীচে এমন শব্দগুলির একটি তালিকা রয়েছে যা ব্যথা এবং সম্পর্কিত লক্ষণগুলির বিভিন্ন গুণাবলীর কিছু বর্ণনা করে। অনুগ্রহ করে সেই সংখ্যাগুলি বৃত্ত করুন যা গত 12 মাসে আপনি যে ব্যথা এবং সম্পর্কিত উপসর্গগুলি অনুভব করেছেন তার প্রতিটির তীব্রতা সর্বোত্তমভাবে বর্ণনা করে । যদি শব্দটি আপনার ব্যথা বা সম্পর্কিত লক্ষণগুলি বর্ণনা না করে তবে 0 ব্যবহার করুন।

| 1. ধক্ধকানি ব্যথা | কোনটিই নয় | 0 | 1 | 2 | 3 | 4 | 5 | 6 | 7 | 8 | 9 | 10 | সবচেয়ে খারাপ সম্ভব |
| --- | --- | --- | --- | --- | --- | --- | --- | --- | --- | --- | --- | --- | --- |
| 2. শুটিং ব্যথা | কোনটিই নয় | 0 | 1 | 2 | 3 | 4 | 5 | 6 | 7 | 8 | 9 | 10 | সবচেয়ে খারাপ সম্ভব |
| 3. ছুরিকাঘাতের ব্যথা | কোনটিই নয় | 0 | 1 | 2 | 3 | 4 | 5 | 6 | 7 | 8 | 9 | 10 | সবচেয়ে খারাপ সম্ভব |
| 4. তীক্ষ্ণ ব্যথা | কোনটিই নয় | 0 | 1 | 2 | 3 | 4 | 5 | 6 | 7 | 8 | 9 | 10 | সবচেয়ে খারাপ সম্ভব |
| 5. ক্র্যাম্পিং  ব্যথা | কোনটিই নয় | 0 | 1 | 2 | 3 | 4 | 5 | 6 | 7 | 8 | 9 | 10 | সবচেয়ে খারাপ সম্ভব |
| 6. কুটকুট ব্যথা | কোনটিই নয় | 0 | 1 | 2 | 3 | 4 | 5 | 6 | 7 | 8 | 9 | 10 | সবচেয়ে খারাপ সম্ভব |
| 7. গরম জ্বলন্ত ব্যথা | কোনটিই নয় | 0 | 1 | 2 | 3 | 4 | 5 | 6 | 7 | 8 | 9 | 10 | সবচেয়ে খারাপ সম্ভব |
| 8. ধরা ব্যথা | কোনটিই নয় | 0 | 1 | 2 | 3 | 4 | 5 | 6 | 7 | 8 | 9 | 10 | সবচেয়ে খারাপ সম্ভব |
| 9. ভারী ব্যথা | কোনটিই নয় | 0 | 1 | 2 | 3 | 4 | 5 | 6 | 7 | 8 | 9 | 10 | সবচেয়ে খারাপ সম্ভব |
| 10. টেন্ডার | কোনটিই নয় | 0 | 1 | 2 | 3 | 4 | 5 | 6 | 7 | 8 | 9 | 10 | সবচেয়ে খারাপ সম্ভব |
| 11. বিভাজন ব্যথা | কোনটিই নয় | 0 | 1 | 2 | 3 | 4 | 5 | 6 | 7 | 8 | 9 | 10 | সবচেয়ে খারাপ সম্ভব |
| 12. ক্লান্তিকর | কোনটিই নয় | 0 | 1 | 2 | 3 | 4 | 5 | 6 | 7 | 8 | 9 | 10 | সবচেয়ে খারাপ সম্ভব |
| 13. বিরক্তিকর | কোনটিই নয় | 0 | 1 | 2 | 3 | 4 | 5 | 6 | 7 | 8 | 9 | 10 | সবচেয়ে খারাপ সম্ভব |
| 14. ভীতিজনক | কোনটিই নয় | 0 | 1 | 2 | 3 | 4 | 5 | 6 | 7 | 8 | 9 | 10 | সবচেয়ে খারাপ সম্ভব |
| 15. শাস্তি-নিষ্ঠুর | কোনটিই নয় | 0 | 1 | 2 | 3 | 4 | 5 | 6 | 7 | 8 | 9 | 10 | সবচেয়ে খারাপ সম্ভব |
