# Supplementary material for: A cross-cultural study translating and validating the COMPAT-SF pain questionnaire in Telugu, Bengali and Hindi
Source: Indian J Gastroenterol. 2025 Feb 18;44(5):684–91. doi: 10.1007/s12664-025-01737-z (PMC12417265; doi:10.1007/s12664-025-01737-z)
Supplement: Supplementary file 3 — Supplementary file3 (PDF 306 KB) [file 12664_2025_1737_MOESM3_ESM.pdf]

क्रोनिक पैनक्रिएटाइटिस (अग्नाशयशोथ) में दर्द के आकलन के लिए व्यापक साधन - संक्षिप्त प्रपत्र (कॉम्पेट-एसएफ)

दिनांक: __________

प्रतिभागी का नाम :	एनआईएच :	लिंग:
जातीयता :	उम्र :	पेशा :

इस अध्ययन में भाग लेने के लिए धन्यवाद। कृपया नीचे अपना विवरण भरें या रोगी का लेबल लगा दें।

निर्देश: निम्नलिखित प्रश्नों में **क्रोनिक पैनक्रिएटाइटिस (अग्नाशयशोथ)** में आपके **पैनक्रियास** (अग्नाशय) में होने वाले दर्द के अनुभव के बारे में पूछा गया है। यह आम तौर पर पेट के ऊपरी हिस्से में कहीं महसूस होता है।

प्रश्न1. कृपया अग्नाशय के दर्द के उस पैटर्न पर गोला बनाएं जो पिछले 12 महीनों के दौरान आपके दर्द के अनुभव के बारे सबसे अच्छी तरह बताता है।

सबसे ज्यादा दर्द ↕ दर्द नहीं	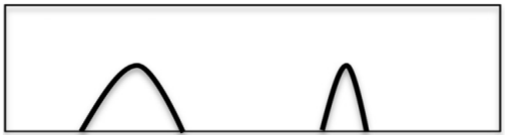 ए. कभी तेज दर्द उठना और बीच में कोई दर्द नहीं होना	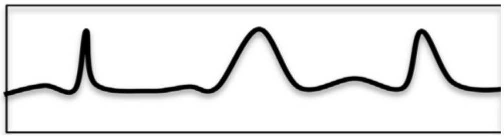 सी. बहुत तेज दर्द उठने के साथ लगातार दर्द होते रहना
	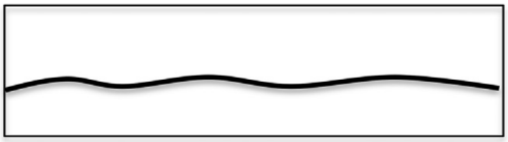 बी. हर दिन लगातार दर्द होते रहना	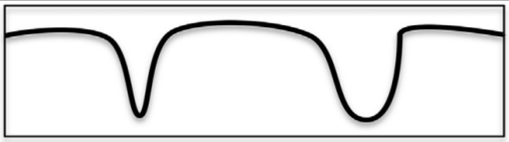 डी. बीच में कुछ कम दर्द के साथ पेट में लगातार दर्द होते रहना

प्रश्न 2. पिछले 12 महीनों के दौरान आपके दर्द की गंभीरता

कृपया इन पैमाने में से प्रत्येक पर X लगाएं

1. दर्द की औसत गंभीरता	0 1 2 3 4 5 6 7 8 9 10<br>दर्द नहीं मध्यम दर्द सबसे ज्यादा दर्द
2. दर्द की सबसे ज्यादा गंभीरता	0 1 2 3 4 5 6 7 8 9 10<br>दर्द नहीं मध्यम दर्द सबसे ज्यादा दर्द
3. दर्द की सबसे कम गंभीरता	0 1 2 3 4 5 6 7 8 9 10<br>दर्द नहीं मध्यम दर्द सबसे ज्यादा दर्द

प्रश्न 3. कृपया अपने दर्द के लिए खाने जाने वाली दवाएं और खुराक नीचे लिखें और आवृत्ति के लिए अपने उत्तरों पर गोला लगाएं

**दवा**	**खुराक**	**आवृत्ति**
		पीआरएन/ओडी/बीडी/टीडीएस/क्यूआईडी
		पीआरएन/ओडी/बीडी/टीडीएस/क्यूआईडी
		पीआरएन/ओडी/बीडी/टीडीएस/क्यूआईडी
		पीआरएन/ओडी/बीडी/टीडीएस/क्यूआईडी
		पीआरएन/ओडी/बीडी/टीडीएस/क्यूआईडी

पीआरएन : जरूरत होने पर, ओडी : दिन में एक, बीडी : दिन में दो बार टीडीएस : तीन बार, क्यूआईडी : दिन में चार बार

प्रश्न 4. कृपया प्रत्येक मद के बारे में बताएं जो आपके पैनक्रियास के दर्द को पैदा करता है

मद	कभी नहीं	बहुत कम	कभी कभार	कई बार	हमेशा	लागू नहीं
1. कोई भी खाना	☐	☐	☐	☐	☐	☐
2. चिकनाई वाला खाना	☐	☐	☐	☐	☐	☐
3. पीने के तरल पदार्थ	☐	☐	☐	☐	☐	☐
4. शराब पीना	☐	☐	☐	☐	☐	☐
5. तनाव	☐	☐	☐	☐	☐	☐
6. सिगरेट पीना	☐	☐	☐	☐	☐	☐
7. कसरत	☐	☐	☐	☐	☐	☐
8. सामाजिक मेल जोल	☐	☐	☐	☐	☐	☐
9. मौसम में बदलाव	☐	☐	☐	☐	☐	☐
10. त्वचा पर हल्के से छूना	☐	☐	☐	☐	☐	☐
11. त्वचा पर ठंडा / गर्म	☐	☐	☐	☐	☐	☐
12. त्वचा पर दबाव	☐	☐	☐	☐	☐	☐
13. अन्य (कृपया बताएं) :	☐	☐	☐	☐	☐	☐

प्रश्न 5. आपके प्रारूपिक पैनक्रियास से संबंधित दर्द के अलावा कृपया आपको होने वाले प्रत्येक अनुभव के मद का मूल्यांकन करें

मद	कभी नहीं	बहुत कम	कुछ बार	अक्सर	हमेशा
1. सिर और/या चेहरे का दर्द	☐	☐	☐	☐	☐
2. जोड़ों का दर्द	☐	☐	☐	☐	☐
3. ऊपरी और /या / निचले अंगों में दर्द	☐	☐	☐	☐	☐
4.पीठ और/या गर्दन में दर्द (अग्न्याशय दर्द से संबंधित नहीं)	☐	☐	☐	☐	☐
5. पेट और/या पेडू में दर्द (अग्न्याशय दर्द से संबंधित नहीं)	☐	☐	☐	☐	☐
6. मांसपेशियों में दर्द	☐	☐	☐	☐	☐
7.सीने में दर्द	☐	☐	☐	☐	☐
8.अन्य : (कृपया बताएं)	☐	☐	☐	☐	☐

प्रश्न 6. नीचे उन शब्दों की सूची दी गई है जो दर्द और संबंधित लक्षणों के कुछ विभिन्न गुणों के बारे में बताते हैं। कृपया उन नंबरों पर गोला बनाएं जो पिछले 12 महीनों के दौरान आपके द्वारा महसूस किए गए दर्द और संबंधित लक्षणों में से प्रत्येक की तीव्रता का सबसे अच्छा वर्णन करते हैं। यदि शब्द आपके दर्द या संबंधित लक्षणों को सही तरीके से नहीं बताता है तो 0 लिखें।

1. धड़कने जैसा दर्द	कभी नहीं	0	1	2	3	4	5	6	7	8	9	10	सबसे खराब संभव
2. तेजी से उठने वाला दर्द	कभी नहीं	0	1	2	3	4	5	6	7	8	9	10	सबसे खराब संभव
3. छुरा घोंपने जैसा दर्द	कभी नहीं	0	1	2	3	4	5	6	7	8	9	10	सबसे खराब संभव
4. तेज दर्द	कभी नहीं	0	1	2	3	4	5	6	7	8	9	10	सबसे खराब संभव
5. ऐंठन का दर्द	कभी नहीं	0	1	2	3	4	5	6	7	8	9	10	सबसे खराब संभव
6. कुतरने का दर्द	कभी नहीं	0	1	2	3	4	5	6	7	8	9	10	सबसे खराब संभव
7. गर्म जलने जैसा दर्द	कभी नहीं	0	1	2	3	4	5	6	7	8	9	10	सबसे खराब संभव
8. दुखी करने वाला दर्द	कभी नहीं	0	1	2	3	4	5	6	7	8	9	10	सबसे खराब संभव
9. बहुत ज्यादा दर्द	कभी नहीं	0	1	2	3	4	5	6	7	8	9	10	सबसे खराब संभव
10. नर्म	कभी नहीं	0	1	2	3	4	5	6	7	8	9	10	सबसे खराब संभव
11. फटने जैसा दर्द	कभी नहीं	0	1	2	3	4	5	6	7	8	9	10	सबसे खराब संभव
12. शरीर में थकान	कभी नहीं	0	1	2	3	4	5	6	7	8	9	10	सबसे खराब संभव
13. बीमार होना	कभी नहीं	0	1	2	3	4	5	6	7	8	9	10	सबसे खराब संभव
14. डर जाना	कभी नहीं	0	1	2	3	4	5	6	7	8	9	10	सबसे खराब संभव
15. बहुत तकलीफ होना - क्रूर	कभी नहीं	0	1	2	3	4	5	6	7	8	9	10	सबसे खराब संभव

आपके भाग लेने के लिए धन्यवाद
